# Supplementary material for: Influence of Two Hexose Transporters on Substrate Affinity and Pathogenicity in Magnaporthe oryzae
Source: Microorganisms. 2024 Mar 28;12(4):681. doi: 10.3390/microorganisms12040681 (PMC11052475; doi:10.3390/microorganisms12040681)
Supplement: Supplementary file 1 [file microorganisms-12-00681-s001.zip › microorganisms-2903835-supplementary.pdf]

Supplementary

Supporting Information Legends

A

B

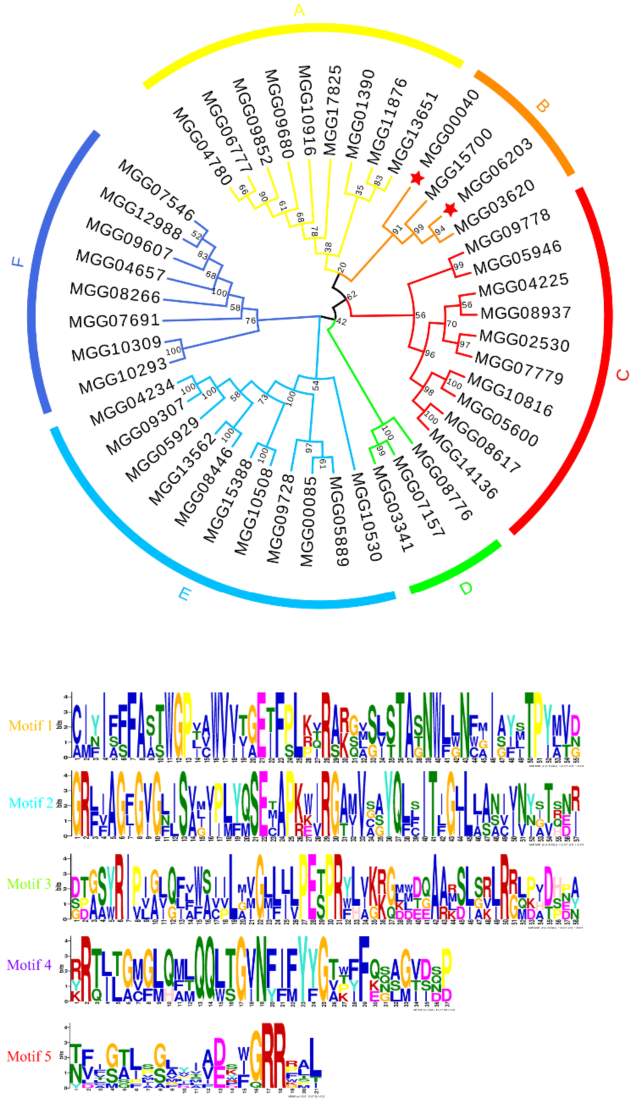

Figure S1. Phylogenetic tree and domain prediction analyses of the *MoHXTs* and their homologs from *Magnorporthe oryzae*. (A) The phylogenetic tree was constructed by MEGA7's Neighbor-Joining (NJ) method, and 1000 replications were performed for bootstrap values. The six clusters of proteins are distinguished by different colors. (B) Domain architectural analysis of Class b. The conserved motifs of MoHXT proteins along with a scale used to estimate the length of the proteins were drawn using TBtools software. Motif 1–6 were indicated. Y-axis values show the best possible matching E-Value.

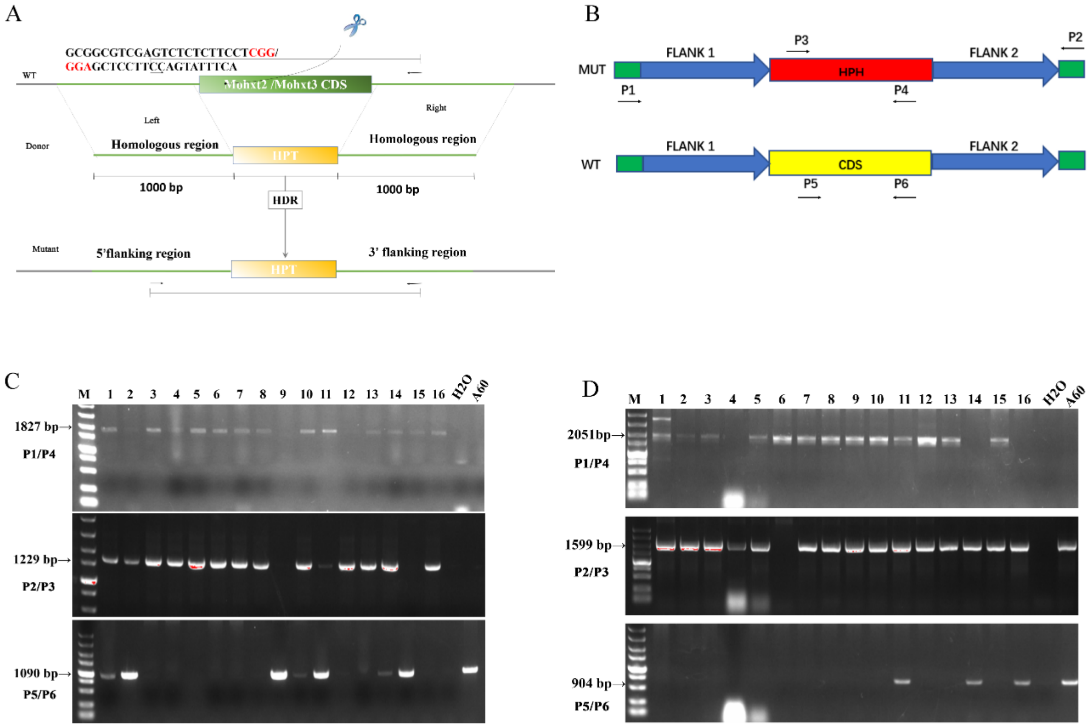

Figure S2. Schematic overview of the targeted gene replacement strategy and PCR diagnostic gel electrophoresis. (A) Illustration of the genomic target sequence for *MoHXT2* targeting the RNP-CRISPR-Cas9 complex, showing a typical double-stranded break next to the protospacer-adjacent motif (PAM) site. (B) Positions of primer used for verification of the mutants. Two homologous arms and the hygromycin region were verified by the primer pairs P1+P4 and P3+P2, and the coding region (CDS) was checked by using the primer pair P5+P6 (primers are listed in Table S4). (C) PCR diagnostic gel for *ΔMohxt2*. (D) PCR diagnostic gel for *ΔMohxt3*.

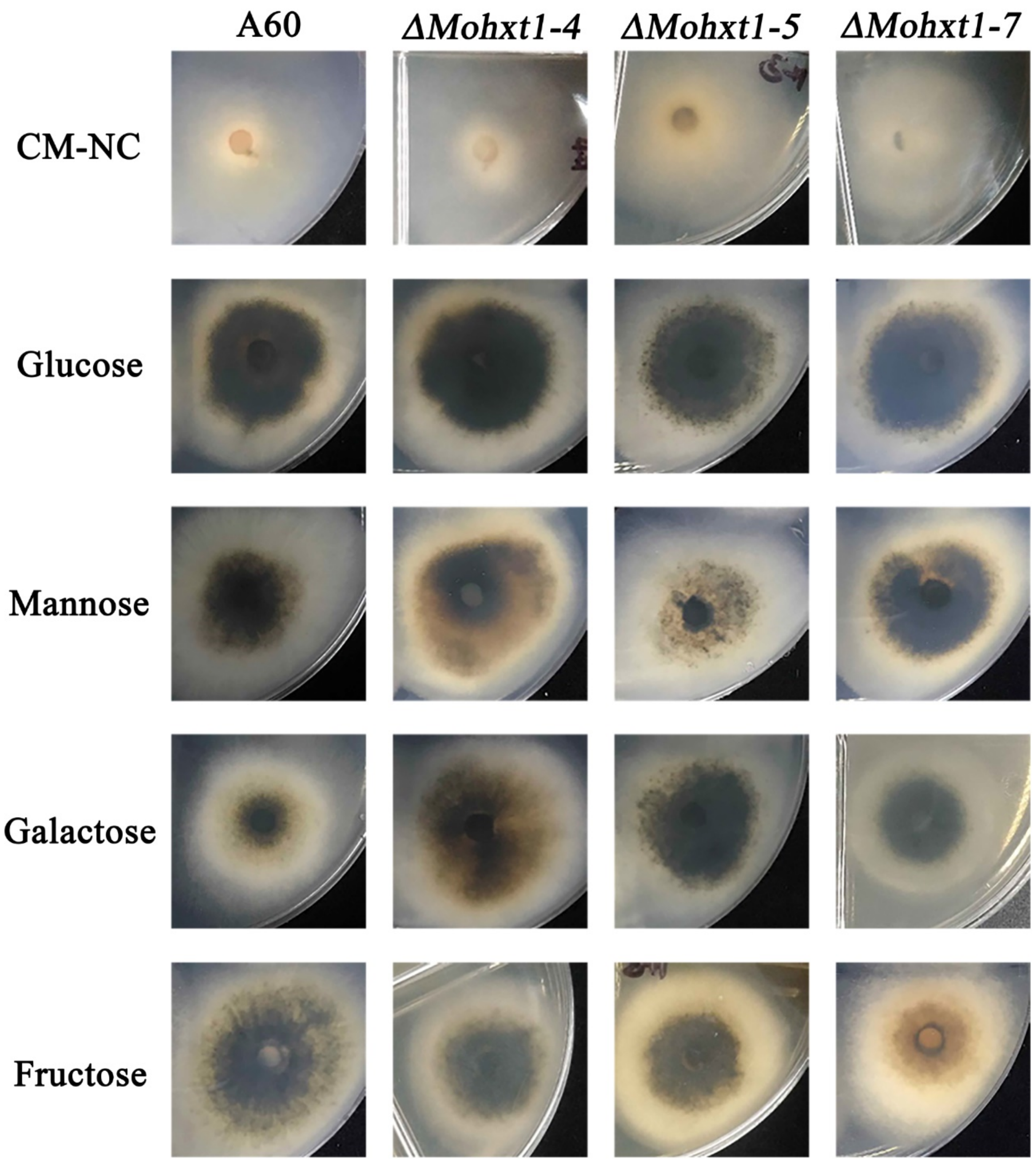

Figure S3. Growth of the restored functionality strains with the gene of *MoHXT2* and *MoHXT3*. Strains were cultivated on CM media supplemented with various carbon sources, including glucose, mannose, or fructose. And a parallel control lacking any carbon source (CM-NC) was included.

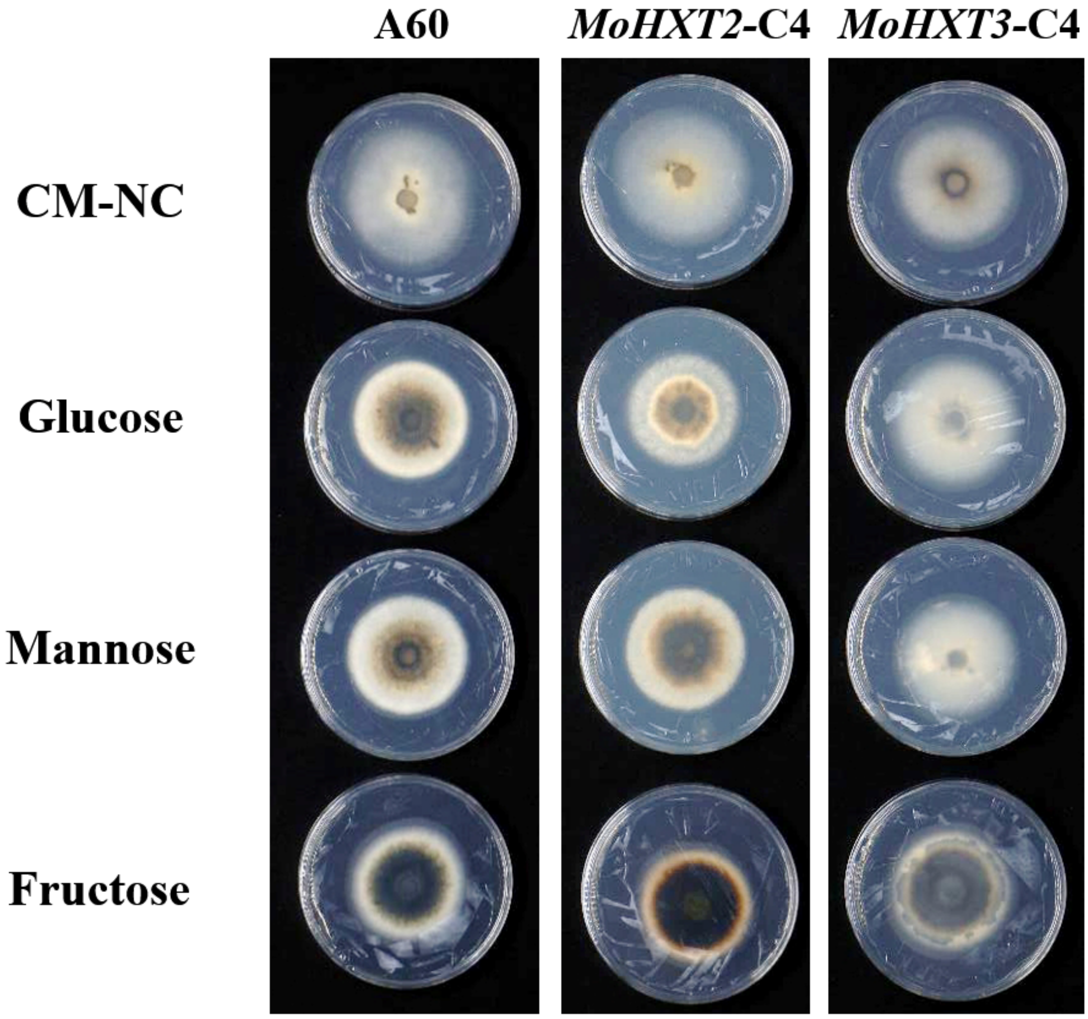

Figure S4. Melanin production of A60 and the *ΔMohxt1* mutant on CM medium supplemented with different carbon sources (glucose, mannose, galactose and fructose). A medium without any carbon source was used as the control (CM-CN).

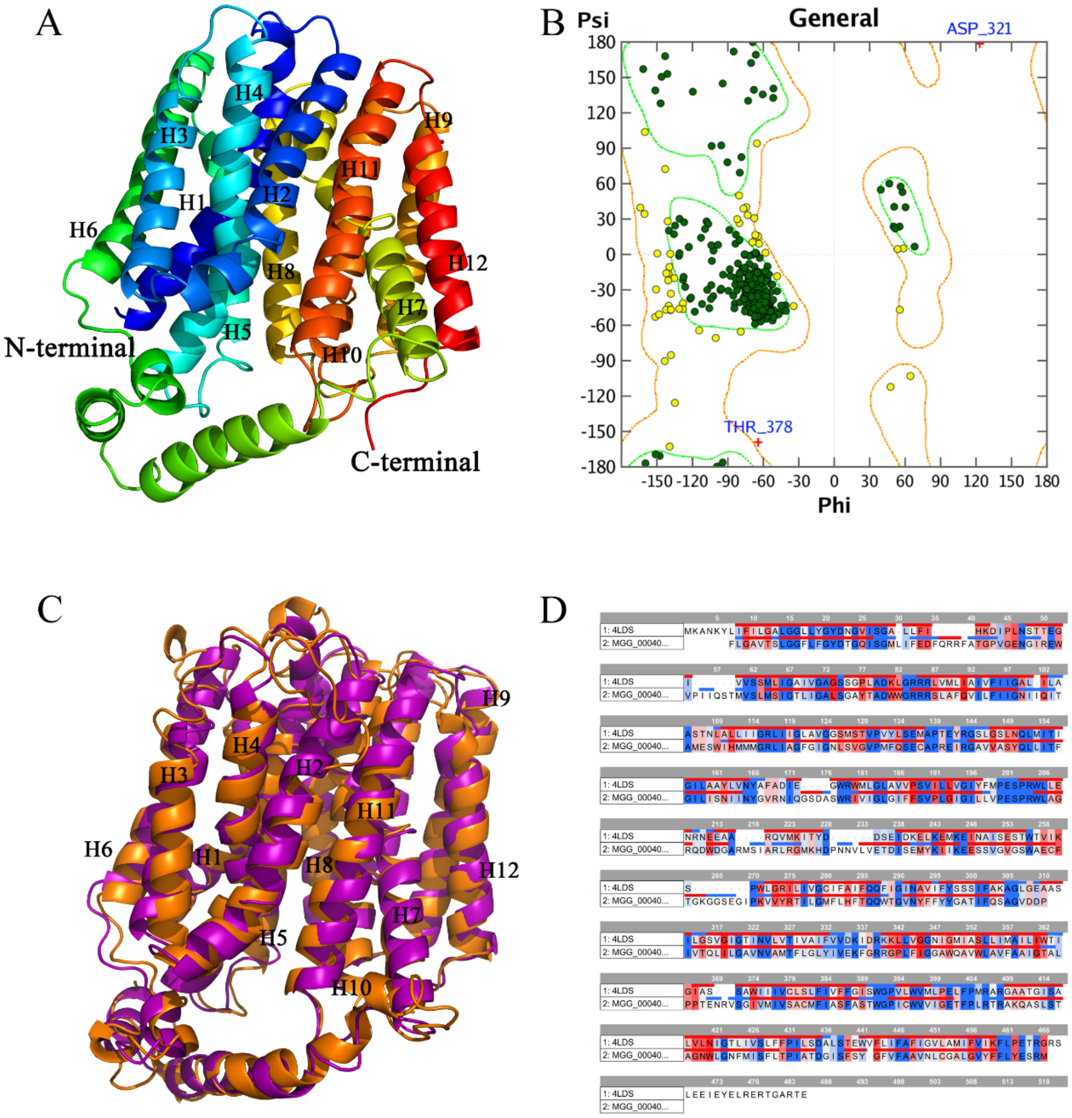

Figure S5. Modeling structural analysis of *MoHXT2*. (A) Homology model of *MoHXT2*. (B) Ramachandran plot for *MoHXT2*. Dark green dots represent the residues in favored regions; yellow dots represent the residues in allowed regions, red cross represent the residues in irrational regions. (C) Superposition of the *MoHXT2* model structure and the template structure. The *MoHXT2* structure is shown in purple, the template structure is shown in orange, and the average RMSD value of the overlay is 1.729 Å. (D) Structure-based sequence comparison between *MoHXT2* and the template structure. The same or similar residues are highlighted in blue, and dissimilar residues are highlighted in red, where the darker colors indicate more similar or dissimilar residues. The sequences corresponding to the alpha-helix are marked by a red line. Template crystal structures were identified through BLAST and downloaded from the RCSB Protein Data Bank (PDB ID: 4LDS).

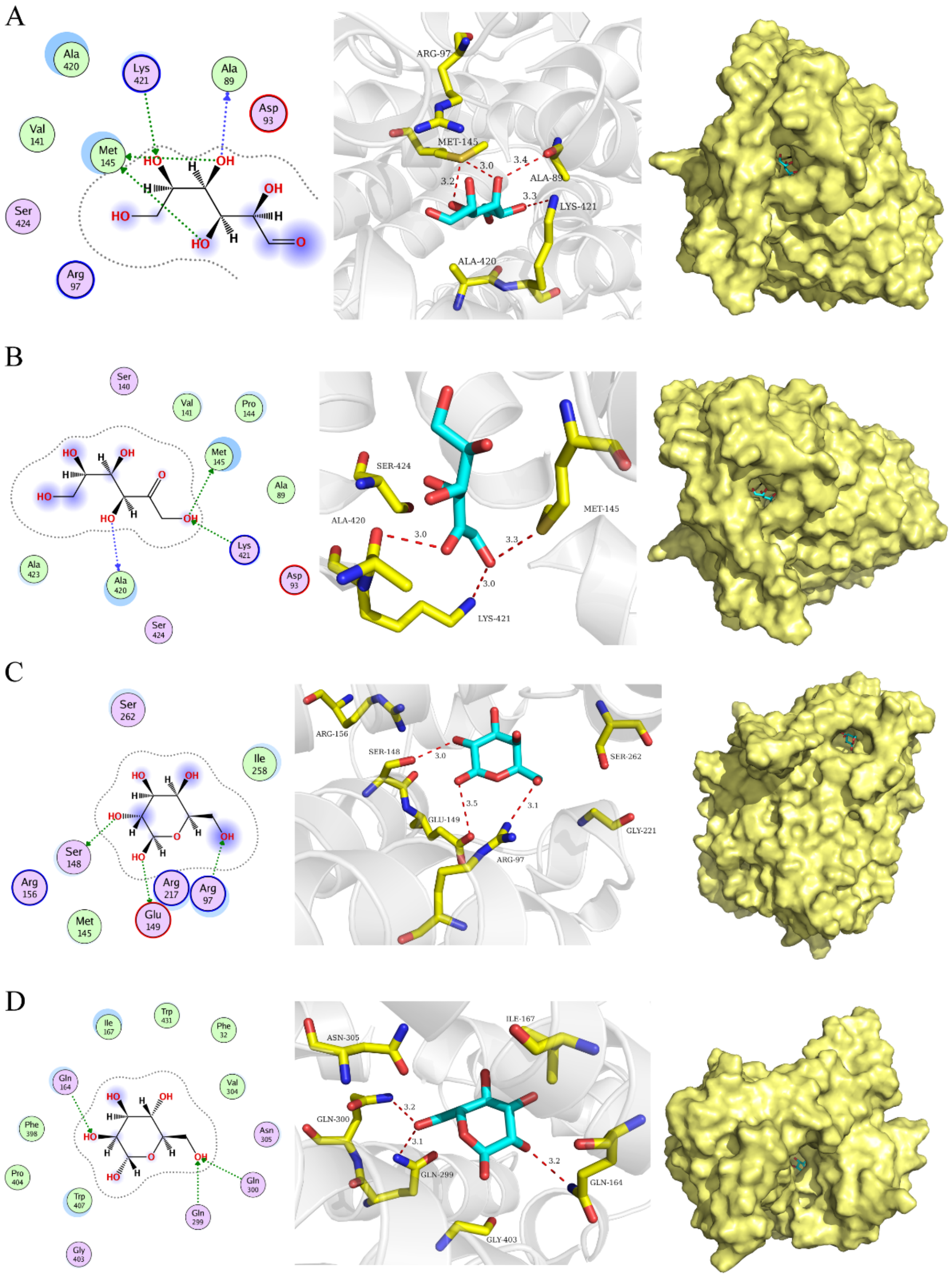

Figure S6. 3D binding model between *MoHXT2* and each hexose. (A) D-(+)-Glucose-50-99-7, (B) D-(-)-Fructose-57-48-7, (C) D-Galactose-59-23-4, and (D) D-Mannose-3458-28-4 are colored cyan. The surrounding residues in the binding pockets are colored yellow. The backbone of the receptor is shown in pale-yellow. The hydrogen bond is depicted as a red dashed line.

Supplementary Tables

Table S1. The structure of 4 monosaccharides

Monosaccharide	Structure
D (+)-Glucose-50-99-7	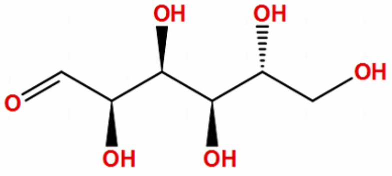
D-(-)-Fructose-57-48-7	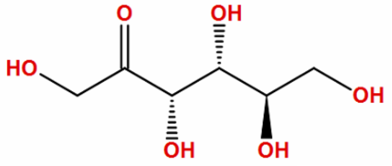
D-Galactose-59-23-4	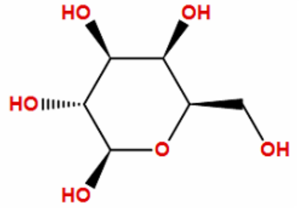
D-Mannose-3458-28-4	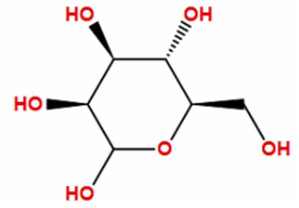
Phlorizin dihydrate	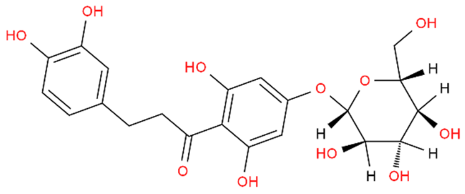

Table S2. The fpkm value of MoHXTs in A60, *ΔMohxt2* and *ΔMohxt3* strains

id	A60_fpkm	MoHXT2_fpkm	MoHXT3_fpkm
MGG_00005	5.11	81.85	78.07
MGG_00015	2.42	4.76	9.17
MGG_00016	10.10	12.72	19.99
MGG_00017	0.89	1.13	5.83
MGG_00018	59.61	94.08	539.23
MGG_00019	128.04	78.70	1743.63
MGG_00020	27.24	25.43	583.84
MGG_00022	0.03	1.91	3.92
MoHXT3	306.28	533.04	11.99
MGG_00032	9.79	13.18	14.66
MGG_00033	102.63	159.39	201.01
MGG_00039	6.90	21.56	11.39
MoHXT2	11.35	1.13	7.22
MGG_00049	21.67	17.42	25.5
MGG_00050	17.37	8.69	17.21
MGG_00052	55.70	220.95	87.82
MGG_00068	278.76	187.82	183.03

Table S3. Plasmids used in this study.

Strains/Plasmids	Description	Sources
Strains		
M. oryzae A60	The wild-type strain	From Yunnan Academy of Agricultural Sciences
ΔMohxt2	*ΔMohxt2*, *MoHXT2* deletion mutant strain	This study
ΔMohxt3	*ΔMohxt3*, *MoHXT3* deletion mutant strain	This study
EBY.VW4000	The *S. cerevisiae* strain is deficient in monosaccharide transport	From Northwest A&F University
Plasmids	Description	Sources
PDR-195	Heterologous expression vectors in *S. cerevisiae*	From Northwest A&F University
PDR-*MoHXT2*	Heterologous expression *MoHXT2* in *S. cerevisiae*	This study
PDR-*MoHXT3*	Heterologous expression *MoHXT3* in *S. cerevisiae*	This study
RP27-eGFP	Direct cloning vector	In lab
RP27-*MoHXT2*	Cloning vector carrying intact *MoHXT2* gene	This study
RP27-*MoHXT3*	Cloning vector carrying intact *MoHXT3* gene	This study
pYF11-GFP	Restored functionality vectors in *M. oryzae*	In lab
pYF11::MoHXT2	Restored functionality *MoHXT2* in *M. oryzae*	This study
pYF11::MoHXT3	Restored functionality *MoHXT3* in *M. oryzae*	This study
pT7TSHA	Heterologous expression vectors in *X. oocytes.*	In lab
pT7TSHA-*MoHXT2*	Heterologous expression *MoHXT2* in *X. oocytes.*	This study
pT7TSHA-*MoHXT2*	Heterologous expression *MoHXT3* in *X. oocytes.*	This study

Table S4. Primers used in this study.

Primer name	Primer sequences (5′– 3′)	Remark
MoHXT3 flank 1F	CCGCTGACATGGACGCCAGAAAG	Construction of homology arm
MoHXT3 flank 1R	TTGGGTTAGTATTGGGATGAGAA	Construction of homology arm
MoHXT3 flank 2F	GATCGAGGGCGACGCCAGCGACCAC	Construction of homology arm
MoHXT3 flank 2R	GCATCAGATTGGCCGCAGATATTAC	Construction of homology arm
MoHXT3CDS F	ATGAGGCCGGTGCCGCCTGGC	Amplify CDS region for complementation assay
MoHXT3CDS R	TTAGACCCTGTTGGCCTGATCG	Amplify CDS region for complementation assay
MoHXT3 sgRNA1 Forward	CCTCTAATACGACTCACTATAGGAGCTCCTTCCAGTATTTCAGTTTAAGAGCTATGC	sgRNA construction
HXT2 flank 1F	CCGCACTTTGTGGCTGCCCCTTGG	Construction of homology arm
MoHXT2 flank 1R	CATTGACCCTGATGGACCTGTAC	Construction of homology arm
MoHXT2 flank 2F	ACGCCAAGCAAAGGCCTGGTAGC	Construction of homology arm
MoHXT2 flank 2R	TCTCCTCCATCACGGGCAGCAAC	Construction of homology arm
MoHXT2 CDSF	ATGCTTGGCGGCAAGTAAGTT	Amplify CDS region for complementation assay
MoHXT2 CDSR	TTACGCGTTCCGGAGCACAGCC	Amplify CDS region for complementation assay
Cleaved gRNA R	ATGAAACCGACACCGAGACCAG	Amplify for Cleaved gRNA
HPT(5′R)	ATAAAGGGAGGAAGGGCGAAC	Amplify HPH gene
HPT (3′ F)	GCGTTTCGGGTTTACCTCTTCC	Amplify HPH gene
MoHXT1 flank 1F	CCACTACCAGGCAAACAAAG	Amplify for homology arm

MoHXT1 flank 1R	AACAGGAGGGAAGGTGAGGC	Amplify for homology arm
MoHXT1 flank 2F	ATCGCCACCGAGTCTGCCAAGGAGG	Amplify for homology arm
MoHXT1 flank 2R	TCAATTTCGTCCGTCTGTACCTG	Amplify for homology arm
MoHXT1CDS F	ATGCCCGGCTCCGTCATCGGG	Amplify CDS region for complementation assay
MoHXT1CDS R	TTAGACGGTCTCCTCCTTGGC	Amplify CDS region for complementation assay
MoHXT1 sgRNA1 Forward	CCTCTAATACGACTCACTATAGGATACCACCAAAGGCGGCAAGTTTAAGAGCTATGC	Amplify for SgRNA
pDR195 F-00040	ACCCCAGCCTCGAGCATGCTTGGCGGCAAGTCCATCA	Yest expression vector
pDR195R-00040	GAAGTCCAAAGCTGGATCTTACGCGTTCCGGAGCACAGCC	Yest expression vector
pDR195F-06203	ACCCCAGCCTCGAGCATGGTTCGCGGATTCAAGGGC	Yest expression vector
pDR195R-06203	GAAGTCCAAAGCTGGATCTTAGACCCTGTTGGCCTGATC	Yest expression vector
pT7-00040-F	GATCTGATATCACTAGTGCCACCATGCTTGGCGGCAAGTCCATCA	Oocyte expression vector
pT7-00040-R	CGCGGCCGCCTCGAGGCATGCTTACGCGTTCCGGAGCACAGCC	Oocyte expression vector
pT7-06203-F	GATCTGATATCACTAGTGCCACCATGGTTCGCGGATTCAAGGGC	Oocyte expression vector
pT7-06203-R	CGCGGCCGCCTCGAGGCATGCTTAGACCCTGTTGGCCTGATC	Oocyte expression vector
DTB-Lys-F	CCGCACGCGTGCCATGCAGGCCT	Site–directed Mutagenesis
DTB-Lys-R	ATGGCACGCGTGCGGAGCGGGAAGGT	Site–directed Mutagenesis
